# Medical Versus Surgical Treatment for the Management of Diabetic Foot Osteomyelitis: A Systematic Review

**DOI:** 10.3390/jcm10061237

**Published:** 2021-03-17

**Authors:** Aroa Tardáguila-García, Irene Sanz-Corbalán, Josep M. García-Alamino, Raju Ahluwalia, Luigi Uccioli, José Luis Lázaro-Martínez

**Affiliations:** 1 Diabetic Foot Unit, Clínica Universitaria de Podología, Facultad de Enfermería, Fisioterapia y Podología, Universidad Complutense de Madrid, Instituto de Investigación Sanitaria del Hospital Clínico San Carlos (IdISSC), 28040 Madrid, Spain; aroa_tg@hotmail.com (A.T.-G.); diabetes@ucm.es (J.L.L.-M.); 2 GhenderS Research Group, Universidad Blanquerna-Ramon Llull, 08022 Barcelona, Spain; josepmariagarciaa@gmail.com; 3 Department of Orthopedics, King’s College Hospital, London SE5 9RS, UK; r.ahluwalia1@nhs.net; 4 Department of Systems Medicine, University of Tor Vergata, Viale Oxford 81, 00133 Rome, Italy; luccioli@yahoo.com

**Keywords:** diabetic foot osteomyelitis (DFO), medical treatment, surgical treatment, systematic review

## Abstract

A systematic review and quality assessment was performed to assess the management of diabetic foot osteomyelitis by medical or surgical treatment. The Preferred Reporting Items for Systematic Reviews and Meta-Analyses (PRISMA) checklist was used. All selected studies were evaluated using the Cochrane Risk of Bias Tool to assess the risk of bias for randomized controlled trials. The literature was revised using PubMed (Medline) and Embase (Elsevier) up to September 2020 to identify clinical trials assessing medical or surgical treatment to manage diabetic foot osteomyelitis. A total of six clinical trials that met our inclusion criteria, with a total of 308 participants. Healing rate, complete closure of the wound, and type of complications were the outcomes evaluated. Risk of bias assessment showed that only two of the six clinical trials included in the systematic review had a low risk of bias. Based on our findings, we believe that the management of diabetic foot osteomyelitis remains challenging. There are few high-quality clinical trials that both stratify clinical presentations and compare these treatments. We conclude that the available evidence is insufficient to identify the best option to cure diabetic foot osteomyelitis.

## 1. Introduction

Diabetic foot osteomyelitis (DFO) is considered a frequent and severe complication of diabetic foot infections (DFIs), affecting approximately 20% of moderate DFIs and between 50% and 60% of severe DFIs [1,2]. DFO can lead to both minor or major amputation and death [3]. DFO represents a challenging diagnostic and management condition; its devastating consequences are related to late diagnosis, late referral, and late and inadequate treatment [2].

There is a growing trend for non-surgical management of DFO [4], which has resulted in good results in remission from infection, with rates being higher than 60% in patients managed exclusively with antibiotic therapy [5]. The published literature is, however, limited to retrospective studies [6,7,8,9,10,11,12], and in certain selected cases, surgery is essential, for instance, where there is bone exposure and/or severe bone destruction, and/or patients with antibiotic resistance or medical treatment failure [2,13]. The surgical approach is based on timely containment of infection with effective debridement [14], utilizing conservative surgical techniques, which aim to avoid unnecessary minor and major amputations [15,16]. The remission rates after surgical treatment are varied, but it has been estimated as ~50% [17,18]. Several studies have concluded that a combination of conservative surgery and antibiotic therapy could be the most appropriate treatment for DFO [15,19,20,21].

Nevertheless, debate continues about the best approach to DFO [13,22,23]. A multidisciplinary approach is mandatory in this type of infection [24]. In 2014, Lázaro-Martínez et al. [25] published the first randomized controlled trial (RCT) to directly compare medical versus surgical treatment, highlighting the importance of combined conservative surgery and antibiotic therapy. Further studies have been reported comparing these treatment options, but are few and are limited by selection criteria. The purpose of this systematic review is to assess the cure rates after receiving medical or surgical treatment in patients with DFO by systematically reviewing the published literature.

## 2. Materials and Methods

The Preferred Reporting Items for Systematic Reviews and Meta-Analyses (PRISMA) checklist [26] was used to perform this systematic review. All selected studies were evaluated with the Cochrane Risk of Bias Tool in order to assess the risk of bias for randomized controlled trials (RCTs) [27].

### 2.1. Literature Search Strategy

All searches were conducted in September 2020. The databases PubMed (Medline) and Embase (Elsevier) were searched to identify clinical trials assessing medical or surgical treatment to manage DFO. The electronic database search was done using the following terms and combinations: “diabetic foot osteomyelitis” AND “treatment”. Studies published in English, Spanish, French, and German were included. To identify additional reports, the reference list of retrieved studies was cross-checked. Abstracts were checked to exclude studies meeting our exclusion criteria, and full texts were reviewed to determine if the studies fulfilled our inclusion criteria. Two authors (ATG and ISC) independently performed the review; any discrepancies between them were discussed with a third author (JLM).

### 2.2. Eligibility and Selection Criteria

The following essential criteria were used to assess the studies:

- The inclusion criteria were clinical trials or randomized controlled trials published in English, Spanish, French, or German, including humans >18 years old, with DFO treated by antibiotics or surgery.
- The exclusion criteria were animal, preclinical, or in vitro studies; non-original papers (review, case report, letter, or comment); and studies with insufficient data for analysis. Furthermore, references to reviews (narrative and systematic) were examined to identify additional articles.

### 2.3. Data Collection

A customized Microsoft Excel spreadsheet was used to extract the data from the studies. The extracted data included: author name, year of publication, design of the study, number of included patients, intervention evaluated and comparison, and outcome measures (healing rate, complete closure of the wound, and type of complications).

### 2.4. Outcome Measures

We extracted patient demographic data, study sample size, and participant/treatment group. The primary outcome measure in this study was the cured rates and their measurement. Secondary outcome measures included: functional outcomes; mortality; post-treatment complications, including re-operation/revision rate between treatment.

### 2.5. Assessment of Study Methodology and Quality

Quality assessment was performed using the Cochrane Risk of Bias Tool for RCTs [27]. Studies that met more than four of the seven criteria set by this tool received a grade of HIGH quality or STRONG evidence.

## 3. Results

### 3.1. Studies

**Figure 1:**
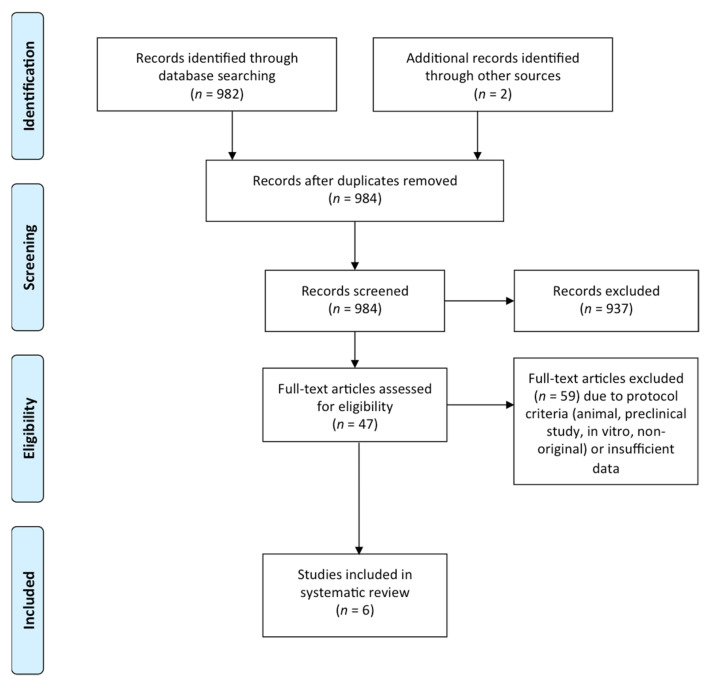
Flow chart of identified studies.

A total of 982 manuscripts were identified from the literature. After screening the title and abstracts, we identified 47 potential records. After screening, a total of six [25,28,29,30,31,32] clinical trials met the selection criteria and were included in this systematic review (Figure 1).

All studies were published between 1994 and 2015, from 13 countries and three journals. RCTs were included in the systematic review, but only one study compared both treatments directly for the management of DFO [25], which was the first RCT comparing surgical versus medical treatment for osteomyelitis.

### 3.2. Patient Characteristics and Identification of Infection

A total of 308 patients were included (range, 21–77 patients per study), with a median sample size of 51.3 ± 19.2 patients. Each study described a unique diagnostic methodology for DFO identification. Confirmation of DFO was based on imaging tests in combination with bone culture, probe-to-bone (PTB), laboratory tests, or clinical evidence of infection. In five of six studies [25,28,30,31,32], imaging included radiographic (*n* = 4) and MRI assessment (*n* = 1) and in some studies was serially undertaken. No formal classification system of osteomyelitis was used in any study.

### 3.3. Treatment and Intervention Strategies

Of the six studies that met the inclusion criteria, antibiotics were used in all the studies, and either used in isolation (*n* = 3) or combined with surgery (*n* = 3). Between 16.7 and 63.4% of patients were treated with antibiotics (median of 39.5 ± 16.6% per study), and 28.8–81.0% (median of 49.9 ± 27.5% per study) of patients were treated with surgery. Only one study provided a direct comparison of medical treatment versus surgical treatment [25]. All studies used a combination of parenteral and/or oral antibiotics, but only one study defined a course as an end-point.

Two studies evaluated the efficacy and safety of two antibiotic treatments in all types of diabetic foot infection, with a specific group of patients with DFO [28,30]. Lauf et al. [28] conducted a Phase 3 trial to compare parenteral tigecycline to intravenous ertapenem, with or without adjunctive vancomycin; in subjects with DFO, they found that the cure rates were low with a 150 mg once-daily regimen of tigecycline. Lipsky et al. [30] compared intravenous and oral formulations of linezolid with that of ampicillin-sulbactam, with linezolid at least as effective as ampicillin-sulbactam. The same authors compared intravenous ofloxacin followed by oral ofloxacin or intravenous ampicillin/sulbactam followed by oral amoxicillin/clavulanate, concluding that each of the therapeutic regimens used can cure or improve most patients [31]. Grayson et al. [32] compared imipenem/cilastatin versus ampicillin/sulbactam, reporting that these treatments are similar.

Two studies [31,32] evaluated the efficacy of two relatively broad-spectrum therapeutic regimens, initially administered parenterally and then orally, but patients who had evidence of osteomyelitis were not enrolled in the study unless all the infected bone was removed. One study defined the main objective [29] as comparing 6-week versus 12-week durations of antibiotic treatment in DFO treated non-surgically. Antibiotics were administered either orally for the entire treatment period or intravenously for a short period (5 to 7 days), followed by a long course of oral antimicrobial therapy.

### 3.4. Treatment Outcomes

The evaluated outcomes differed amongst the studies. One study [29] evaluated remission of DFO; four studies registered clinical response as the principal outcome [28,30,31,32], and one assessed healing rates [25]. Clinical response was considered as the primary end-point in three studies included in the review.

The most frequent complication reported was drug-related events (*n* = 12/40; 30%). The most frequent complications registered were re-infections (*n* = 11/92; 12%), amputations (*n* = 8/92; 8.6%), and death (*n* = 4/114; 3.5%). Patients requiring reintervention were rare. Table 1 summarizes the data extracted from the six selected studies. Three studies reported higher rates of re-infection and amputations and death rates in antibiotic-only treatment groups [25,28,29].

### 3.5. Assessment of Study Bias

**Figure 2:**
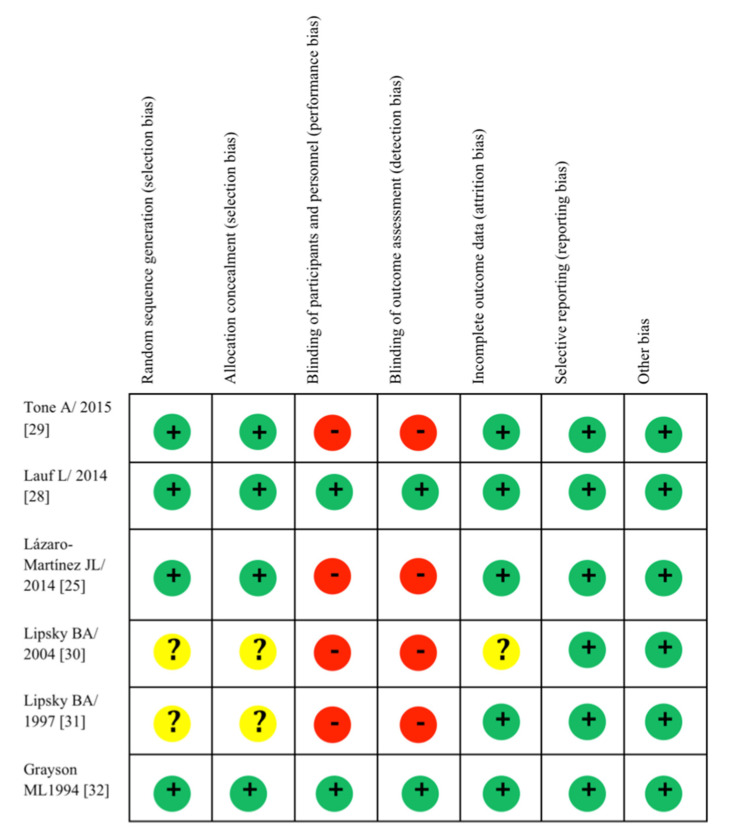
Quality assessment (Cochrane Risk of Bias tool) for included RCTs. A green circle with a plus sign indicates a low risk of bias, a yellow circle with a question mark indicates an unclear risk of bias, a red circle with a minus sign indicates a high risk of bias.

The risk of bias assessment of the six RCTs included in the systematic review is collectively given in Figure 2, separated for each research question. Two of the studies reviewed demonstrated a LOW risk of bias, with two displaying SOME CONCERNS, and two demonstrating HIGH risk (Figure 2).

## 4. Discussion

### 4.1. Diagnostic Methods of DFO

All studies included in this systematic review utilized different methods to diagnosis DFO, contributing to the heterogeneity between groups. This variability in the diagnostic methodology promotes a limitation in comparability due to study selection criteria. Although the gold standard of DFO is positive histopathology with concordant microbiology results by bone biopsy, and the institution of culture-specific antibiotics, this is not universally reported. This is probably because the majority of the RCTs have evaluated antibiotic treatments, where a bone biopsy could not or was not performed to follow a non-invasive treatment strategy.

Radiographs were used in most studies (*n* = 4) to aid in diagnosing DFO. The combination of the PTB test and radiography has a sensitivity and specificity similar to magnetic resonance imaging (MRI) for the diagnosis of DFO (0.97 of sensitivity and 0.92 of specificity). MRI has high usage in DFO diagnosis with high sensitivity and specificity (0.90 and 0.83, respectively) [33], and plain radiography on its own has a low sensitivity (0.54) for the diagnosis of DFO [34]. However, when these tests are used in isolation, without correlation with clinical characteristics, agreement on the diagnosis of DFO is low [35,36].

For this reason, it is more appropriate if the clinician examines the ulcer beforehand, using a combination of a clinical test, imaging test, and bone biopsy to make the final diagnosis more reliable [37]. At the present time, there is no consensus on the role of specific laboratory markers for the diagnosis in DFO, and again, these should be considered with a combination of other clinical tests [2,38,39].

### 4.2. Treatments for DFO

The lack of homogeneity in the treatments applied in the studies complicates attempts to perform meta-analyses and to analyze the rates of DFO remission or complication between two types of treatments. The type and administration of antibiotic treatment differed among all the studies evaluated. Two studies applied prior surgical debridement as a prerequisite to the selection and entry into the respective study, and only one study compared two treatment groups directly (surgical versus antibiotic) [25]. Two studies evaluated the efficacy and safety of two antibiotic treatments in all types of diabetic foot infection, with a specific group of patients with DFO [28,30].

### 4.3. Ulcer Healing Versus DFO Cure

It is difficult to compare an antibiotic treatment alone with surgical treatment as the study goals might be similar, but the end-points utilized are different. The main outcomes described include a clinical response with remission of inflammatory signs, where the on-going bone infection is not demonstrated or thought to be clinically eradicated [15].

Ideally, the criteria to define DFO remission should be based on a direct measure of infection from bone culture and histology. Most of the current literature [6,9,40,41,42] defines DFO remission as wound healing, avoiding recurrent ulceration, amputation, recurrent infection, or any combination of these. It has been shown that DFO remission is not directly related to these surrogate markers/measurements and is not specifically associated with remission of osteomyelitis [43]. Most of the studies [28,30,31,32] considered the main outcome to be a clinical response and categorized the resolution of all clinical signs and symptoms of infection after treatment as “cured”.

Only two studies considered the main outcome as ulcer healing, where patients were followed up for at least 12 weeks after healing [25,29]. Additionally, Tone et al. [29] considered DFO remission being the stabilization or improvement in radiographic abnormalities on plain X-rays assessed at the end of treatment and 1 year later. It is important to consider that the DFO might not be curable, and remission might be what is pragmatically achievable. Armstrong et al. [44] proposed that it might be more useful to think of patients who have achieved wound closure as being in remission rather than being cured. Independent predictors of recurrence of foot ulcers include plantar ulcer location and the presence of osteomyelitis [45].

For this reason, considering only clinical response as a measure of the cure for a DFO might only identify patients who are considered to be in remission and not as fully healed or cured. Today, there is still no defined follow-up period when the clinically healed patient can be considered to have the DFO resolved. The authors would recommend follow up after ulcer closure for at least 1 year [39].

### 4.4. Rates of DFO Remission

The overall percentage of patients with DFO remission is greater in those patients undergoing surgical treatment. However, there is a wide variation in observed remission rates between studies (28.8–81.0%). It is difficult to draw an inference or conclusion, other than identifying the aforementioned trend in DFO management. Clearly, there are confounding variables (e.g., pressure ulceration and surgical removal would also aid remission, which would not be dealt with by medical therapy on its own). In addition, after healing, patients need to be evaluated periodically because there are also precipitating factors that led to ulcer recurrence. The first response is peripheral neuropathy that should be managed using preventive methods [44]. Therefore, adequate treatment options, according to the specific characteristics of the patient, are important, as well as considering the timely initiation of treatment in assessing remission and reporting [23,37].

### 4.5. Complications (Short Term and Long Term)

Only three of the included studies describe the short-term or immediate complications in DFO patients, and none report long-term complications. The most frequent complication registered was re-infection, amputation, and two studies reported death rates at 1 year. Tone et al. [29] identified a high number of re-infections and amputations in their study in patients receiving antibiotic therapy alone. Lázaro et al. [25] and Lauf et al. [28] had similar trends in death rate in their samples within the antibiotic group of patients.

Therefore, it is important to develop RCTs comparing both treatments for the management of DFO and monitor long-term complications. In a recent study [39], the authors report that 73.3% of the patients developed complications during the first year of follow-up after suffering from DFO. In this study, the authors did not compare complications according to the treatment applied, thus losing an opportunity to present important information comparing both treatment and a finite assessment of these complications associated with DFO management. This would be important in the selection and consenting of patients who will more than likely need long-term treatment management, which might include re-intervention [24].

### 4.6. Quality Assessment

All studies analyzed in this systematic review have applied a good RCT methodology. However, a high risk of bias in blinding is noted in two studies (Figure 2). This can be difficult as subjects undergoing surgery will clearly have post-operative stigmata (e.g., wounds), whereas subjects who receive antibiotics will not. Blinding of two groups of different antibiotic treatments or with two different doses would be easier, but again, it would be more difficult to blind for the duration of two different treatments.

### 4.7. Limitations, Strengths, and Weaknesses of the Study

To our knowledge, this is the first systematic review of controlled clinical trials addressing the treatment of DFO. An exhaustive search of the literature and assessment of the quality of those RCTs included was conducted as part of the review. The lack of RCTs available that compare two groups of treatment (surgery versus antibiotics), specifically in patients who suffer from DFO, make it impossible to perform a meta-analysis or network meta-analysis, whilst the heterogeneity of the studies included in the review also precludes this type of analysis.

### 4.8. Unanswered Questions and Future Directions

More high-quality RCTs, which directly compare all forms of surgery versus medical treatment for the management of DFO, in stratified condition and patient groups, are required to improve the knowledge of both treatments. RCTs with only DFO patients will be needed to draw accurate inferences and help understand the advantages and disadvantages of various treatments.

More recently, further questions have been raised about the use of antibiotics, in terms of route and timing. In the case of confirmed osteomyelitis, recent trials have investigated the efficacy of oral-only regimens [46]. Again, these studies focus on a combined surgical and medical approach, and the influence of this combination should also be ascertained in DFO. A standardized outcomes methodology to avoid performance and detection bias and longitudinal analysis will need to be conducted to gauge the efficacy of treatment to cure or remission length.

Many infected ulcers show biofilm-producing bacteria, which are resistant to antibiotics. Typically, we manage bacterial biofilm in chronic wounds by ulcer debridement (sharp, hydrosurgical, or ultrasound), negative pressure wound therapy, and antimicrobials. Nowadays, new strategies have been developed, such as hyperbaric oxygen therapy or selective inhibitors of the detrimental matrix metalloprotease-9 [47,48].

## 5. Conclusions

The available evidence is insufficient to identify the best option to “cure” DFO. Efficient treatment of a DFO might involve a combination of both treatment modalities tested here and require selection of the appropriate method according to the indication and specific characteristics of the patient.

To date, there is a lack of studies directly comparing surgical and antibiotics regimens. Therefore, prospective RCTs are required to develop guidelines for the appropriate management of DFO, defining the role of antibiotics and surgery (debridement, essential conservative surgery, and prophylactic surgical treatments) in the patient work-up.

## Figures and Tables

**Table 1:** Characteristics of studies included in the systematic review.

Author/ Year	Number of Participants	Number of Participants by Group Intervention	DFO Diagnostic Method	Intervention	% Treatment with Antibiotic	% Treatment with Surgery	Outcome	Complications (%)
Tone A/2015 [29]	40	20	Culture of transcutaneous bone biopsy	Antibiotic	40.4%	-	Remission of DFO. Stabilized or improved X-rays. Complete healing of the wound.	Major amputation: 10.0% Reinfection: 27.5% Drug-related events: 30.0%
Lauf L/2014 [28]	62	38	MRI or bone biopsy	Antibiotic	40.3%	-	Clinical response at the test-of-cure visit. Microbiologic efficacy	Death: 3.2%
Lázaro-Martínez JL/2014 [25]	52	25	PTB and plain X-ray	Antibiotic or surgery	36.5%	28.8%	Healing rate. Time to healing. The need for surgery in the antibiotic group. The need for re-operation in the surgery group. Rate of amputation, recurrence, re-ulceration and death.	Re-infection: none Re-ulceration: 11.5% Minor amputation: 7.7% Required reintervention: 5.8% Death: 3.8%
Lipsky BA/2004 [30]	77	57	Laboratory and plain radiography, additionally imaging tests or bone biopsy	Antibiotic	63.4%	-	Clinical response at the test-of-cure visit.	-
Lipsky BA/1997 [31]	21	16	Clinical, laboratory, and plain radiograph findings	Antibiotic and surgery	16.7%	40.0%	Clinical response. Microbiological response.	-
Grayson ML/1994 [32]	56	32	Histopathology findings or radiological or clinical evidence	Antibiotic and surgery	-	81.0%	Clinical response. Microbiological response.	-

Abbreviations: DFO, diabetic foot osteomyelitis; MRI, magnetic resonance imaging; PTB, probe-to-bone.

## Data Availability

The data are available previous request to corresponding author.
